# Forest baseline and deforestation map of the Dominican Republic through the analysis of time series of MODIS data

**DOI:** 10.1016/j.dib.2015.06.006

**Published:** 2015-06-24

**Authors:** Florencia Sangermano, Leslie Bol, Pedro Galvis, Raymond E Gullison, Jared Hardner, Gail S. Ross

**Affiliations:** a Graduate School of Geography and Clark Labs, Clark University, 950 Main St. Worcester, MA 01610, United States; b Rescan Environmental Services, 1111 West Hastings Street, 15th Floor, Vancouver, BC, Canada V6E 2J3; c Hardner and Gullison Associates, 15 Woodland Drive, Amherst, NH 03031, United States; d Barrick Gold Corp. Brookfield Place, TD Canada Trust Tower, Suite 3700, 161 Bay Street, Toronto, Canada M5J 2S1; e Pueblo Viejo Dominicana Corporation, Piso 16, Avenida Lope de Vega No. 29 Ensanche Naco, Santo Domingo, Dominican Republic

**Keywords:** Forest cover, Forest change, Dominican Republic, MODIS

## Abstract

Deforestation is one of the major threats to habitats in the Dominican Republic. In this work we present a forest baseline for the year 2000 and a deforestation map for the year 2011. Maps were derived from Moderate Resolution Imaging Radiometer (MODIS) products at 250 m resolution. The vegetation continuous fields product (MOD44B) for the year 2000 was used to produce the forest baseline, while the vegetation indices product (MOD13Q1) was used to detect change between 2000 and 2011. Major findings based on the data presented here are reported in the manuscript “Habitat suitability and protection status of four species of amphibians in the Dominican Republic” (Sangermano et al., Appl. Geogr.,) [7].**63**, 2015, 55–65

**Table:** Specifications table

Subject area	Geography
More specific subject area	Land cover change mapping
Type of data	Mpk map package (ArcGIS)
How data was acquired	Processing of satellite images
Data format	Processed
Experimental factors	NA
Experimental features	NA
Data source location	Country: Dominican Republic
Data accessibility	The analyzed data is with this paper

Value of the data

• The data presents a recent analysis of deforestation in the Dominican Republic.
• The maps are useful for the identification of deforestation rates across the Dominican Republic.
• The maps are useful for the for the identification of deforestation hotspots to target protection enforcement and forest management.

## 1 Data, experimental design, materials and methods

### 1.1 Baseline delineation of forest in the year 2000

Moderate Resolution Imaging Radiometer (MODIS) vegetation continuous fields (VCF) percent tree cover data (MOD44B) at 250 m resolution was used to define a baseline forest map for the year 2000. MOD44B represents a sub pixel characterization of global vegetation, identifying the proportion of the pixel that has tree cover. A land cover map of the Dominican Republic for the year 2003 (provided by the Dominican Republic Ministry of Environment) was used to derive the appropriate thresholds to define forest. In order to extract the appropriate percent tree cover that would separate forest from non-forest in the Dominican Republic, we intersected the MODIS vegetation continuous fields (VCF) percent tree cover data with the land cover map and used the average percent tree cover within densely forested areas as the threshold. Although the average percent tree cover was 48%, the threshold was rounded up to 50% to produce a more conservative definition forest [7].

**Fig. 1:**
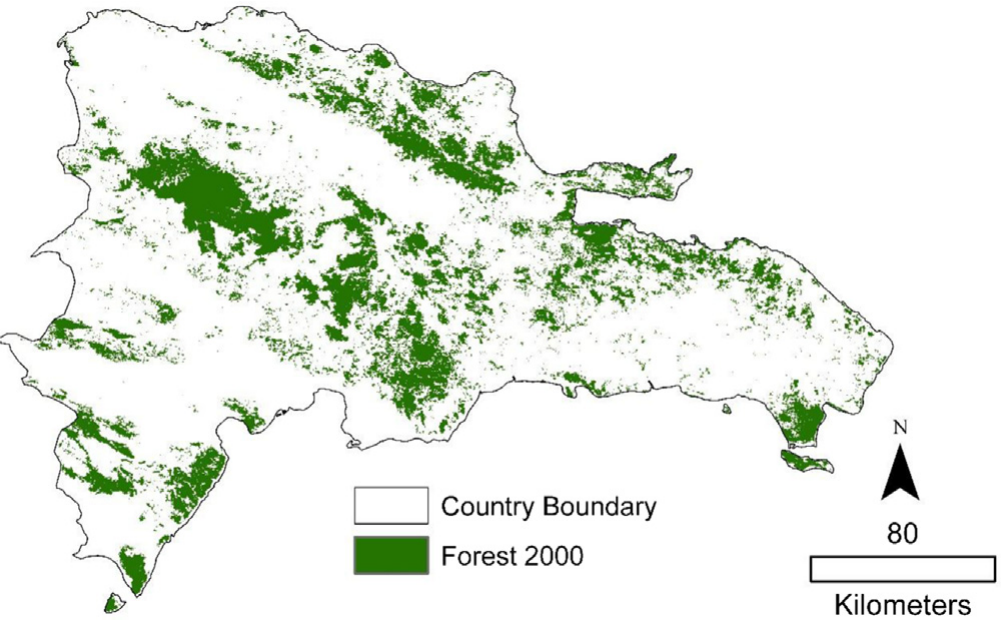
Dominican Republic forest cover in the year 2000.

The forest cover map (Fig. 1) was validated with Google Earth. One hundred points were sampled and a pixel was considered correctly classified as forest if the area of the coarse MODIS pixel contained (visually) more than 50% forest, and was classified as non-forest if the forest area was less than 50%. When possible, past images were used through the historical image option within Google Earth in order to validate using a period of time closer to the date being classified (2000). The forest cover map yielded an accuracy of 86% correct for the forest category, and 74% correct for the non-forest category, with an overall accuracy of 80% correct [7].

### 1.2 Evaluation of changes in productivity of forests

Satellite remote sensing is playing an increasing important role in conservation, in order to map land cover and land uses, to assess vegetation and habitat changes and as inputs in species distribution models [4,5].

Vegetation indices, such as the normalized difference vegetation index (NDVI) allow the evaluation of changes in vegetation quality and quantity. NDVI is measured as the difference in reflectance between the red and the near infrared (NIR) divided by the sum of the reflectance in both bands. Comparison of reflectance in the red and NIR bands is important to evaluate vegetation vigor. Vegetation pigments absorb energy in the visible part of the electromagnetic spectrum, with all pigments absorbing in the blue (0.45 μm) part of the spectrum, and chlorophyll also absorbing in the red (0.65 μm). Moreover, leaf cell structure response in the near infrared (NIR, 1.1–1.3 μm) is characterized with high reflectance. Vegetation stress affects both the absorption in the red (decrease absorption due to change in pigment concentration) and the reflectance in the NIR (decrease reflection due deterioration of the cell structure). Changes in vegetation indices values across time may therefore reflect variations in the quality of the vegetation cover. Pixels in a satellite image do not always represent a homogeneous cover and in many cases, especially when working with coarse spatial resolution datasets, the pixels reflectance values represent a mixture of different land cover types. Exposed soils reflect more red energy and less NIR energy than vegetation, because of this, changes in NDVI values across time can also be due changes in amount of vegetation cover. A decrease in the amount of forest or decrease vegetation vigor within a pixel will cause NDVI values to decrease, while increases in forest cover or vegetation vigor within a pixel, will result in higher NDVI values. Locations with significant declines in NDVI can correspond to both deforestation and forest degradation, while increases in productivity may reflect a within-pixel increase in forest cover (reforestation) or increases in productivity due to increase in, for example, nutrient availability.

### 1.3 NDVI pre-processing: quality filtering

In order to evaluate trends in productivity in areas identified as forest in the year 2000 (from the forest map baseline), we obtained the MODIS vegetation indices time series product (MOD13Q1) from 2001 to 2011. This product contains the enhanced vegetation index, normalized difference vegetation index (NDVI) and quality bands (QC) at 250 m spatial resolution and 16 days temporal resolution. We worked with both the NDVI and QC bands. MODIS QC bands from 2001 to 2011 were used to filter the data to keep only high quality, not contaminated pixels. The quality band for each NDVI image was evaluated, and a pixel was considered reliable if the Mandatory QA flag (bit 0–1) was “VI produced good quality” (value 00). If the mandatory QA flag was “VI produced but check other QA” (value 01), the VI usefulness (bits 2–50) was used to evaluate the quality, and the 3 highest quality values (value 0000, 0001, 0010) were considered reliable [6]. After removing unreliable pixels, a temporal linear interpolation was performed to fill missing data values across time using the Earth Trends Modeler [1] within Idrisi Selva. We only interpolated data gaps if less than three consecutive images (1.5 months) were missing, in order to decrease temporal interpolation artifacts. A filling technique was employed to spatially interpolate speckled missing values, where the value of a missing pixel was filled with the average value of its neighbors only if all its neighbors presented data of reliable quality.

In order to decrease the effect of clouds in the calculation of trends, the NDVI time series was aggregated from 16 days to annual using the maximum value composite approach, where the pixel value of the resulting image is the maximum value across all 23 images within a year. The result is a time series of cleaned maximum NDVI with one image per year.

### 1.4 NDVI trends

**Fig. 2:**
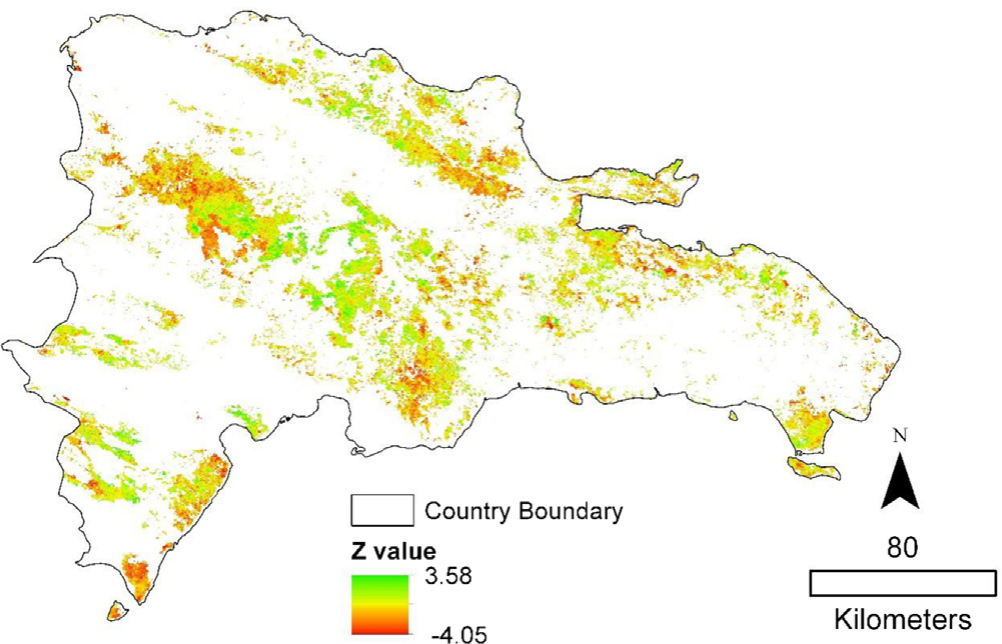
Trend significance. Mann-Kendall *Z*-values. Values of zero have been made transparent.

**Fig. 3:**
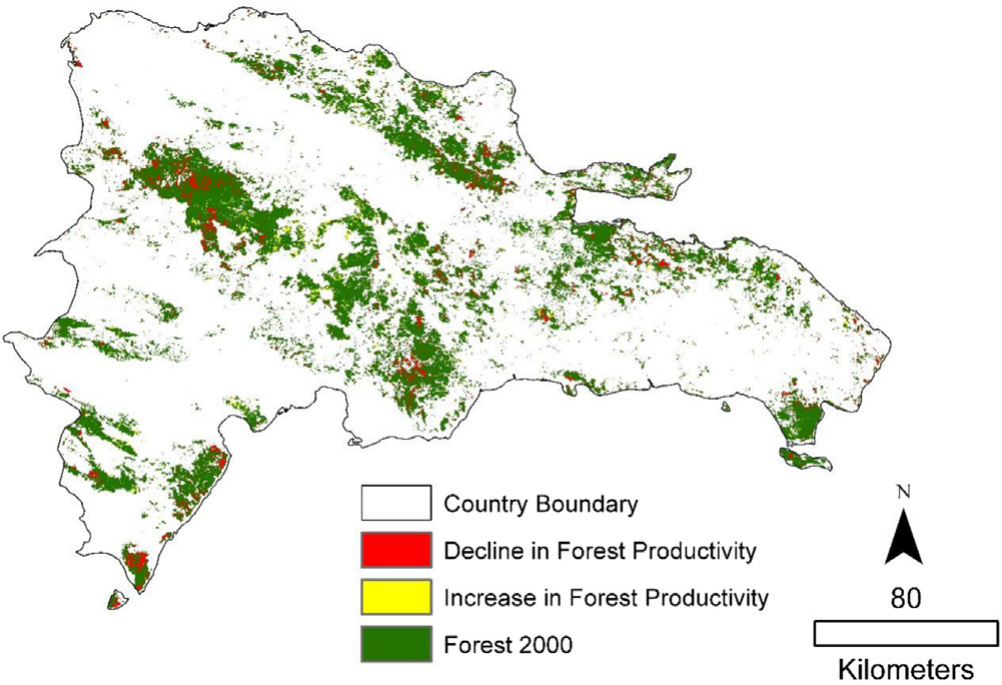
Areas with significant declines and increases in forest productivity at a 95% confidence level.

The 11 years time series of NDVI was clipped to the forest cover baseline in order to only evaluate trends in productivity within areas defined as forest in the year 2000. Trends in the annual NDVI time series from 2001 to 2011 were calculated with the non-parametric Mann–Kendall [3,2] statistic. Significance of the trends was calculated using the Mann-Kendall *Z*-value which follows a normal distribution with mean of zero and variance of 1 under the null hypothesis of no trend. Areas with significant positive trends at a 95% confidence interval were considered deforestation Figs.2–3.

## 2 Processed data in supplementary files

ArcGIS map packages (mpk) are provided for the three figures shown above.

1. Forest2000.mpk: Forest cover for the year 2000. Boolean image.
2. Trend Significance.mpk: Raw Mann Kendall trend significance of NDVI from 2001 to 2011. *Z*-values.
3. Forest Change.mpk: Areas with significant positive or negative trends. Categorical image. 1=decrease in productivity 2=increase in productivity.

## Disclosure statement

The authors understand the journal׳s policy and identify the following potential conflicts of interests. Funding to this research was provided by Pueblo Viejo Dominicana Corporation (PVDC), though a contract to Hardner & Gullison, and subcontract to Clark Labs. P.G, and G.S.R. previously contracted to PVDC and Rescan Environmental Services are now directly employed by PVDC and Barrick Gold Corp., respectively. T.G. and J.H. are partners at Hardner & Gullison.
